# *Madurella mycetomatis* infection of the buttock in an Eritrean refugee in Switzerland: a case report

**DOI:** 10.1186/s13256-018-1962-y

**Published:** 2019-02-12

**Authors:** Carine Mekoguem, Cécile Triboulet, Alexandre Gouveia

**Affiliations:** 1 Department of Ambulatory Care and Community Medicine of the University of Lausanne, Lausanne, Switzerland; 2 Department of Dermatology of the University Hospital of Lausanne, Lausanne, Switzerland

**Keywords:** *Madurella mycetomatis*, Mycetoma, Actinomycetoma, Eumycetoma, Primary care, Migrant health

## Abstract

**Background:**

Mycetoma is a neglected infectious disease caused by a fungus (eumycetoma) or bacteria (actinomycetoma); it is characterized by chronic local inflammation with sinus formation and purulent discharge. Its course can be quite devastating because of the difficulty in diagnosing the infection and in eliminating the causative agent. Although endemic in many countries in the tropics and subtropics, the migration of Africans to Europe may increase the presence of this neglected disease in European countries. We present a case of an Eritrean patient living in a non-endemic country who was diagnosed as having an infection of *Madurella mycetomatis* in an atypical location in his body.

**Case presentation:**

We report the case of a 35-year-old African male refugee from Eritrea, living in Switzerland since 2015, who presented with a 1-year history of a painful soft tissue swelling associated with dark nodules in his right buttock. He mentioned having several previous surgeries after 2001 while he was in Eritrea due to recurrent abscess formation on this body area. In the previous months, the swelling had become more significant and nodules started draining a purulent fluid. An initial diagnostic hypothesis of buttock abscess was made and he was referred to a dermatologist for diagnostic confirmation and further specialist care due to the size and atypical presentation. After a punch biopsy, the diagnosis of eumycetoma was confirmed and cultures developed *Madurella mycetomatis*. The initial treatment approach consisted of oral treatment by itraconazole; however, a surgical resection of the lesions was finally needed.

**Conclusions:**

Although rare, mycetoma should be diagnosed as early as possible to avoid long-lasting complications. Primary care physicians in European countries are frequently in the first line of care of migrant patients and therefore should be aware of the common and uncommon clinical presentations of mycetoma.

## Background

Mycetoma is a chronic subcutaneous infectious disease usually found on the extremities. The infection can be caused by a variety of bacteria (actinomycetoma) and fungi (eumycetoma). In more than 80% of all cases, the foot and legs are affected [1, 2]. Eumycetoma of the hand, buttocks, or thorax is unusual, as seen in the case we describe. The first description of a case of mycetoma is usually attributed to Dr John Gill who reported “Madura foot” in a dispensary report of the Madras Medical Service of the British Army in India, in 1842 [3]. Mycetoma is endemic in many countries in the tropics and subtropics, although most cases are reported from Sudan, Mexico, and India [4]. It occurs typically in young men between the age of 20 and 40 years, especially farmers who are exposed to contaminated soil, and is transmitted during one or more generally minor injuries [1].

In 2013, the World Health Organization added mycetoma to the list of neglected diseases in order to raise awareness of the need for better monitoring and to fight this disease. However, fungal mycetoma is no longer fatal; in the past, the diagnosis often resulted in amputation of limb segment, especially in cases of late diagnosis.

There is scarce literature regarding this infection and there are very few published cases of mycetoma by *Madurella mycetomatis* in Switzerland in the medical literature [5, 6]. This case illustrates one patient with eumycetoma of the buttock, an atypical location, who was treated in the Department of Ambulatory Care and Community Medicine of the University of Lausanne and in the Department of Dermatology of the University Hospital of Lausanne.

## Case presentation

A 35-year-old African male refugee from Eritrea arrived in Switzerland in 2015, after several months of a migratory route through Sudan, Libya, and Italy. This former member of the Eritrean military left his country and exiled himself in Switzerland because of the Eritrean political conflict and for personal security. He is married and has three children in good health. One year after his arrival and during a routine appointment with his primary care physician, he complained of a soft and slightly painful tissue swelling in his right buttock, localized on a previous scar. He mentioned that in 2001 in Eritrea he submitted to surgery several times for recurrent abscess on his right buttock. He was otherwise in good health, had no tobacco smoking or drinking habits, and no regular treatment.

**Fig. 1:**
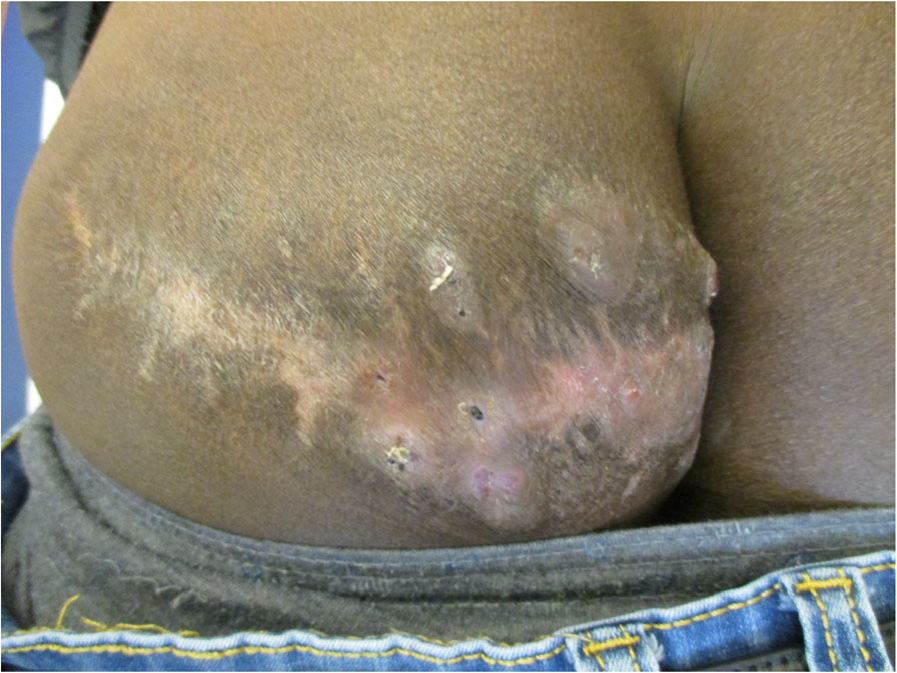
Initial clinical presentation of the buttock with marked swelling and dark exophytic nodules

On physical examination, he had a visible scar approximately 20 cm on the lateral side of his right buttock. On the medial level, the presence of deep indurated exophytic nodules with some visible openings and spontaneous drainage were noted, which suggested an abscess (Fig. 1). He was afebrile and no lymphadenopathy was found.

**Fig. 2:**
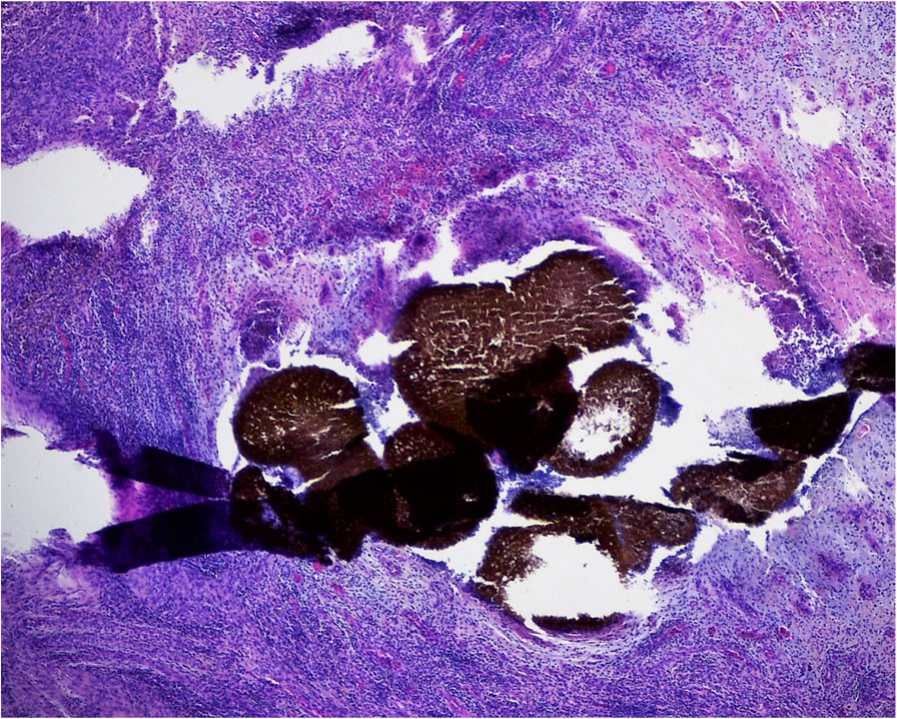
Hematoxylin and eosin stain showing clusters of mycelial filaments and spores in a mixed inflammatory reaction

**Fig. 3:**
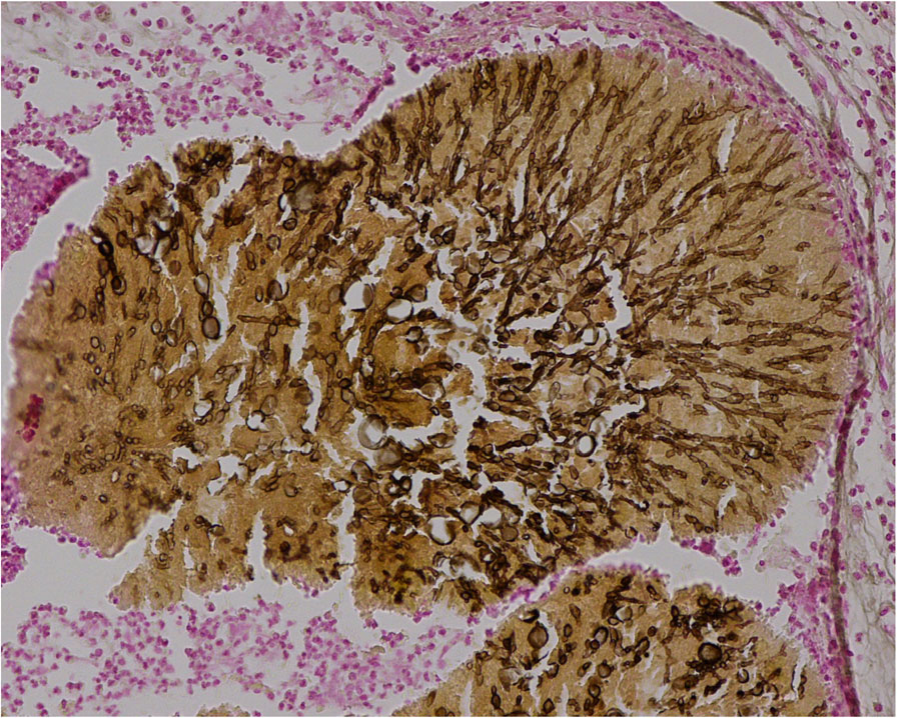
Grocott staining which highlights spores and mycelial filaments

He was referred to the Department of Dermatology at the University Hospital of Lausanne for further investigation. A punch biopsy was performed and during that procedure a sanguinolent discharge was witnessed with conglomerates of small and rather firm blackish pellets, evoking eumycetoma. Tissue and black grain samples were sent for biological and histological evaluation. These revealed chronic suppurative inflammation in the presence of histologic fungal aspects (Figs. 2 and 3). The infectious agent could not be determined exactly at that time, however, the black colored grains indicated a probable *Madurella mycetomatis* infection. A second biopsy was needed and these samples were negative on bacterial culture and positive for fungal culture of *Madurella mycetomatis*, which grew in 2 weeks.

**Fig. 4:**
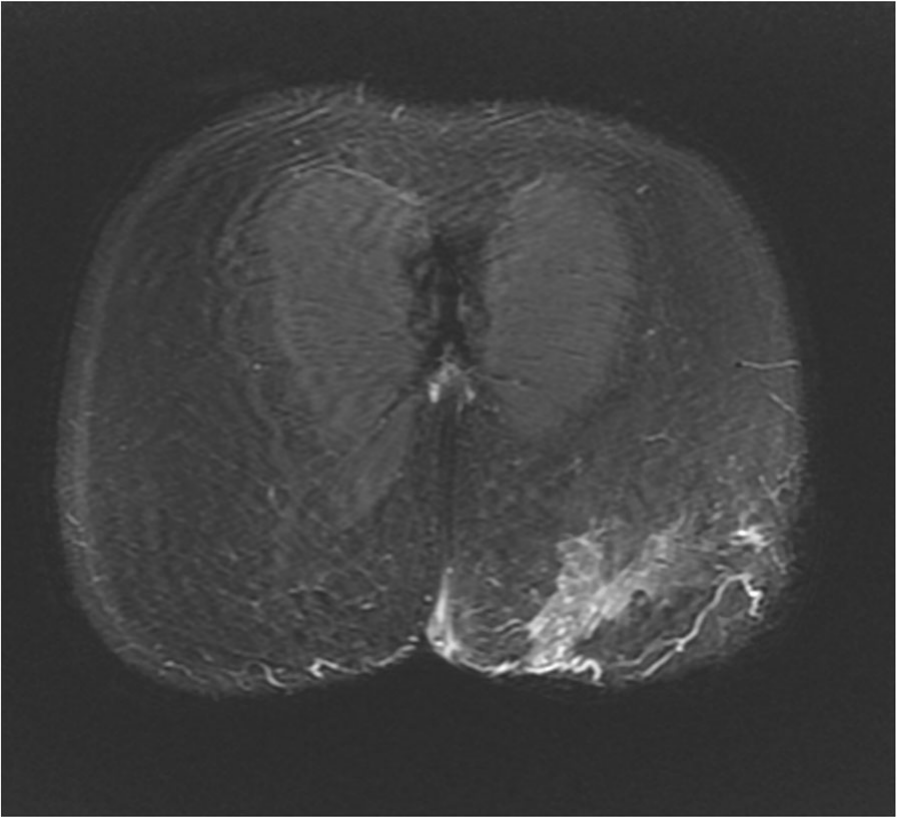
T1-weighted fat-saturated post-contrast magnetic resonance imaging showing pseudo-tumoral infiltration of the subcutaneous fat tissue in the left gluteal region (10 cm × 1.35 cm). Although the muscular plan is slightly involved, the infiltration does not reach a bony structure

**Fig. 5:**
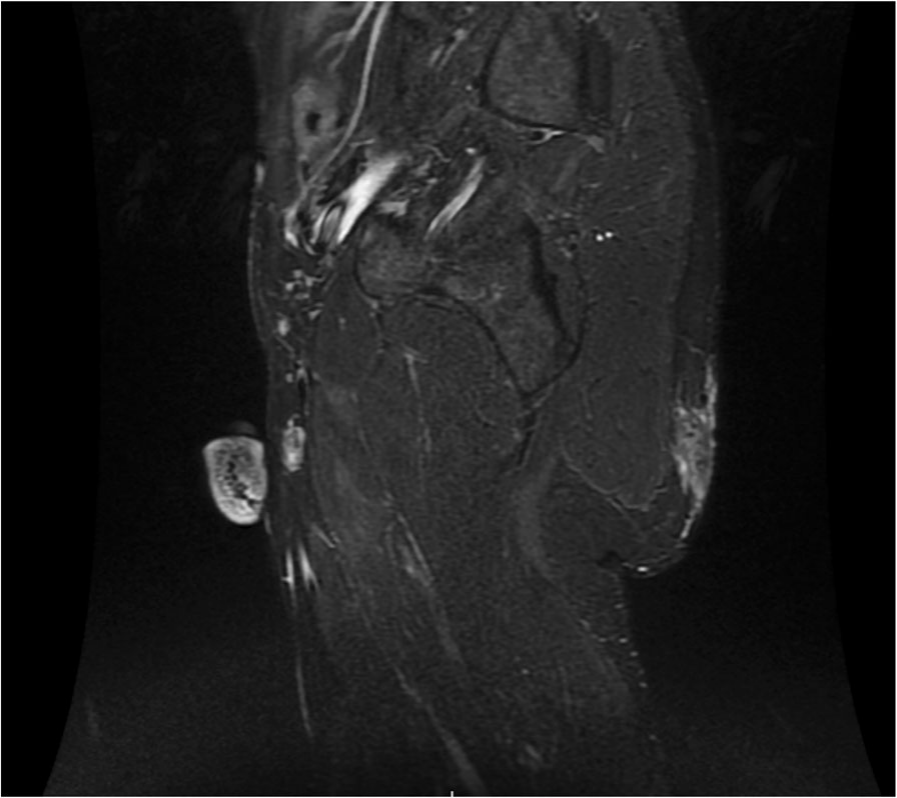
T1-weighted fat-saturated post-contrast magnetic resonance imaging showing pseudo-tumoral infiltration of the subcutaneous fat tissue in the left gluteal region (10 cm × 1.35 cm). Although the muscular plan is slightly involved, the infiltration does not reach a bony structure

To determine the precise depth of the buttock lesions, a magnetic resonance imaging examination of his pelvic area was performed. This examination identified a pseudotumoral infiltration of the cutaneous and subcutaneous tissue into the gluteal muscular plane of the paramedian part of his left buttock compatible with a mycetoma without bone extension (Figs. 4 and 5).

We considered several differential diagnoses of mycetoma such as: recurring epidermal cyst of gluteal region, hidradenitis suppurativa (Verneuil’s disease), pilonidal cyst, cutaneous tuberculosis, profound mycosis, leprosy, and cutaneous metastatic lesions.

**Fig. 6:**
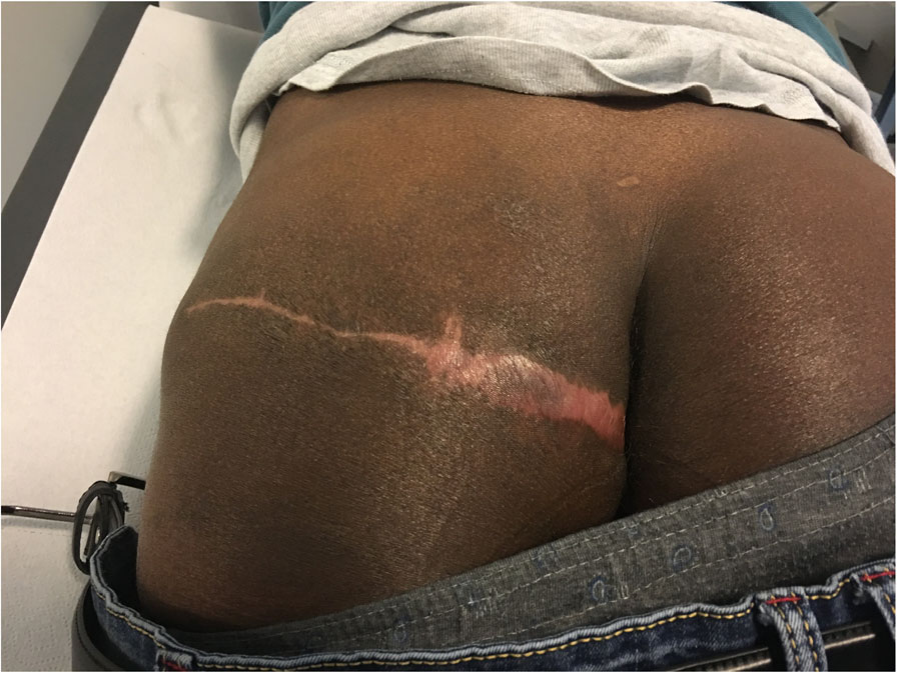
Final result of the pharmacologic and surgical treatment, 3 months after the debridement

He was initially treated with orally administered itraconazole at a dosage of 400 mg once daily for 3 months, which was well tolerated. After antifungal therapy, the lesions did not improve substantially and he had surgical debridement of the lesions followed by a double cutaneous-subcutaneous transposition flap (Fig. 6).

After surgical removal of the lesions, treatment with itraconazole was continued to achieve a total length of 6 months. An ultrasound of his buttock was performed 3 months after the surgery and two residual mycetomatic nodules were identified: one situated at the surgical scar with a size 0.7 × 0.6 cm; and a second one at the intergluteal region with a size of 0.8 × 0.45 cm. Both remained asymptomatic and he did not develop any further lesions at 12 and 24 months after surgery.

## Discussion and conclusions

To the best of our knowledge, this is the first reported case of imported eumycetoma of the buttock, an unusual location, caused by *Madurella mycetomatis* in Switzerland. The recent immigration from countries such as Eritrea and Ethiopia that are close to mycetoma-hyperendemic areas, for example Sudan, increases the probability for European health care professionals to encounter this neglected disease. Health care professionals need to be aware of this entity and suspect mycetoma in patients who present with tumor-like swelling, especially when the patient is a young man and the lesion involves a lower extremity, although not exclusively in this location. A high index of suspicion for mycetoma is needed, and appropriate sampling of the lesion that includes the mycotic grains. An individualized treatment, whether alone or combining pharmacological and surgical approaches, is of increased importance to avoid lesion progression to an advanced and disabling disease.

Mycetoma is usually painless in the first phase of the infection, which can delay diagnosis quite considerably from months to many decades. The diagnosis is even more difficult if the location is atypical and needs to be made by anatomopathology of the excision piece for adequate identification of the causative agent. Once the microbe is inside the human body, it is organized in more or less compact hyphal or hypha-like structures called grains. The grains are widely diverse in shape, size, texture, and color, depending on the etiologic agent. Bacterial grains are usually soft and brittle, with a fine substructure and with colors ranging from off-white to pinkish, whereas fungal grains are rather firm and are composed of microscopically recognizable hyphae, which are either black or whitish. The most common agents of black-grain mycetoma are classified in the genus *Madurella*, such as *Madurella mycetomatis* and *Madurella grisea* [1, 4].

Although rare as an import pathology, mycetoma must be detected early to avoid complications; the main complication is the bone, which is more frequent in actinomycetoma due to it being more osteophilic than fungal agents. Secondary bacterial infections are the frequent late complications of mycetoma; lesions can increase pain and lead to disability or sepsis that can be fatal in the absence of treatment. In addition, bone destruction leads to loss of function in the absence of treatment and is part of the late complications of mycetoma that can lead to amputation. Other complications that can occur include lymphatic obstruction or secondary bacterial infection, osteopenia, and osteoporosis.

There are several tests to diagnose mycetoma. First, the direct microscopic examination of fresh grains between blade and lamella is simple, and it often makes it possible to distinguish fungal grains from actinomycotic grains. The size, the color, and the consistency of the grains, the presence of cement-like material, and the peripheral reaction made by the club bodies should be noted. Actinomycotic grains consist of filaments with a diameter of less than 1 μm; the fungal grains of filaments have a diameter of 2 to 5 μm, often with vesicles [2]. Second, an anatomopathological examination is particularly indicated when the patient is seen at a stage of non-productive fistulas. It also makes it possible to establish diagnosis of the atypical forms, such as the non-fistulized form.

Finally, the new techniques of medical imaging are particularly interesting in the assessment of mycetoma extension. Bone involvement is the major complication and must be systematically investigated by radiology. Mycetoma has characteristic ultrasonographic features, hence, ultrasound imaging appears to be useful in medical centers where no mycological tests can be done [6, 7]. It is also an accurate technique to delimit the extent of the process. Computed tomography or magnetic resonance imaging are more sensitive than ultrasonography to assess the extent of mycetoma in soft tissues and can detect early bone involvement.

This article reports an imported eumycetoma caused by *Madurella mycetomatis* infection of the gluteal region in an Eritrean patient living in Switzerland. In February 2017, 14,927 refugees from Eritrea were in the process of asylum requirement, making it the biggest refugee community in Switzerland [8]. Although data regarding the burden of mycetoma in Eritrea are quite scarce, the geographic proximity to Sudan should raise awareness of this neglected tropical disease that is now appearing in European countries [5, 7]. The close collaboration between primary care physicians and dermatologists was important in diagnosing the eumycetoma infection in this patient, since this infection was not initially considered by the family doctor. Although migrant patients can experience difficulty in accessing health care in several countries, this was not the case for this patient, who had a refugee status and consequent health insurance, as well as an interpreter during the medical appointments.

## Authors’ information

CM is currently a resident in Endocrinology. CT is a resident in Dermatology. AG is a chief resident in General internal medicine. All the authors work currently at the Lausanne University Hospital. AG also works at the Department of Ambulatory Care and Community Medicine of the University of Lausanne.
